# Fine Gold Fillings

**Published:** 1852-04

**Authors:** 


Fine Gold Fillings.—We had the pleasure a few days since of examining a very bad set of teeth most beautifully preserved, (for we consider them preserved) for if such fillings fail to save teeth, we shall despair of success in the plugging of teeth.

The fillings embraced almost every variety of cavity. Some large approximal fillings in the lower molars particularly attracted our notice; we were pleased with the fine space obtained with the file, which not only gave room for manipulation, but gave also a solid and firm border to the wall of the cavity, which we like to see, and these had been well and smoothly trimmed, so that the finish on the gold around the border of the plug was most perfect. The foil had been well consolidated or the fine finish could not have been put on the filling.

The fillings alluded to were inserted by Dr. J. S. Clark of New Orleans, for a young gentleman formerly of this city, on his return from California. We do not recollect the number of plugs, but we believe the Dr. received over $100 for his services, and the gentleman is satisfied he has the worth of his money.

The Dr. in filling teeth rolls his gold into cylinders to suit the size and depth of cavity, we believe very much in the way we prepare what we call blocks for our plugs. The manner of introducing these may be somewhat different, and we believe we are disposed to give him more credit for labor in finishing than we usually take, and yet we regard this as of no small importance. We give him also credit for getting larger fees in New Orleans than we can in Cincinnati. This however, is all right, and we do not think a good dental operation faithfully done in every particular is really dear at any regular price now paid. We are much pleased with the Dr.'s method of filling fangs, and hope he will ere long give our readers a detailed account of his manipulation in filling teeth.

We would merely say that the dental foramen is prepared for filling with broaches prepared with barbs, and that the gold is carried in also on these, and we believe kept in by straps of whalebone as the broach is withdrawn, and thus piece after piece is introduced until the cavity is filled.

We saw not long since a very similar operation as it regards number of plugs and excellent workmanship, by Dr. Hale of St. Louis, Mo. This was for a young lady, and her gems of pearl were truly set with gold.

We think we are not saying too much when we venture the remark that dental operations in the West and South are fast approaching a perfection which will creditably compare with those of the East. We wish only this rivalry, which is, who shall excel.

Our correspondent A. F. E., who gives us the case of transplanting the six front inferior teeth in a gentleman's mouth, by a Philadelphia dentist, is informed that we do not believe the story. A little observation would detect if they were human or sheep's teeth. And we hope he will examine carefully the teeth of sheep and these teeth next opportunity, and satisfy himself, or see if the poor man does not occasionally *blate* like a sheep. If so, we give up the case, and say it is an improvement on Hunter.

Our exchange list will receive attention next number. Such will please direct Dental Register, Cincinnati, Ohio.

